# 

**Published:** 1907-08

**Authors:** 


### CONTENTS.

ORIGINAL COMMUNICATIONS—
Should the Appendix be Removed in All Cases Where the Abdomen is Opened for Other Purposes? By Dr. L. C. Fischer, Atlanta, Ga....257
How the Registration of Nurses Would Benefit the Nurses and Physicians, as Well as the Public. By Mrs. A. C. Hartridge, Atlanta, Ga....263
Care of the Consumptive. By Louis C. Rouglin, M. D., Atlanta, Ga....267
Operation for Hemorrhoids. By William S. Goldsmith, M. D., Atlanta, Ga....275
Discussion of Dr. Goldsmith's Paper....277
The Doctor as a Drug Habitue. By Dr. C. C. Stockard, Atlanta, Ga....280
Puberty and Menapause. By F. H. Johnson, M. D., Ensley, Ala....284

EDITORIAL NOTES AND COMMENTS—
Prohibition and the Doctors....288

NEWS AND NOTES....292

BOOK REVIEWS....295

SELECTIONS AND ABSTRACTS—
Gastric and Duodenal Ulcer....296
The Coated Tongue....296
Latent Diphtheria....297
A Study of Five Hundred Cases of Pleurisy....297
Indicanuria, Its Aetiology and Practical Significance....298
The Influence of Nicotine, Coffee and Tea on the Digestion....299
The Early Diagnosis of Perforated Gastric Ulcer....299
The Beneficent Work of the Surgeon....300
Cardiac Dilatation....301
Nature and Treatment of Piles....302
Transmissibility and Curability of Cancer....303
Amoebic Dysentery....304
Medical Experiences: The Doctor....305
The Diagnosis of Pyelonephritis....306
Pneumonia in Children....306
Organization of the American Medical Association....308
Acute Articular Rheumatism....319

